# Novel Pyoverdine Inhibitors Mitigate *Pseudomonas aeruginosa* Pathogenesis

**DOI:** 10.3389/fmicb.2018.03317

**Published:** 2019-01-09

**Authors:** Daniel R. Kirienko, Donghoon Kang, Natalia V. Kirienko

**Affiliations:** Department of BioSciences, Rice University, Houston, TX, United States

**Keywords:** *Pseudomonas aeruginosa*, antivirulence, siderophore, pyoverdine, *Caenorhabditis elegans*

## Abstract

*Pseudomonas aeruginosa* is a clinically important pathogen that causes a variety of infections, including urinary, respiratory, and other soft-tissue infections, particularly in hospitalized patients with immune defects, cystic fibrosis, or significant burns. Antimicrobial resistance is a substantial problem in *P. aeruginosa* treatment due to the inherent insensitivity of the pathogen to a wide variety of antimicrobial drugs and its rapid acquisition of additional resistance mechanisms. One strategy to circumvent this problem is the use of anti-virulent compounds to disrupt pathogenesis without directly compromising bacterial growth. One of the principle regulatory mechanisms for *P. aeruginosa*’s virulence is the iron-scavenging siderophore pyoverdine, as it governs in-host acquisition of iron, promotes expression of multiple virulence factors, and is directly toxic. Some combination of these activities renders pyoverdine indispensable for pathogenesis in mammalian models. Here we report identification of a panel of novel small molecules that disrupt pyoverdine function. These molecules directly act on pyoverdine, rather than affecting its biosynthesis. The compounds reduce the pathogenic effect of pyoverdine and improve the survival of *Caenorhabditis elegans* when challenged with *P. aeruginosa* by disrupting only this single virulence factor. Finally, these compounds can synergize with conventional antimicrobials, forming a more effective treatment. These compounds may help to identify, or be modified to become, viable drug leads in their own right. Finally, they also serve as useful tool compounds to probe pyoverdine activity.

## Introduction

Despite advances in antimicrobial chemotherapy, multidrug resistant bacteria continue to cause life-threatening infections, especially in hospitals and with immunocompromised patients. *Pseudomonas aeruginosa* is a particularly pernicious pathogen as it possesses several innate defense mechanisms against antibiotics. For example, *P. aeruginosa* expresses four major efflux pumps (MexAB-OprM, MexCD-OprJ, MexEF-OprN, and MexXY-OprM) that effectively reduce intracellular drug concentrations, preventing the compounds from reaching intracellular doses required for effect (Lomovskaya et al., 2001). These pumps confer resistance to several classes of commonly used drugs, including β-lactams, fluoroquinolones, and aminoglycosides (Poole et al., 1993, 1996; Köhler et al., 1997; Aires et al., 1999). Chemically inhibiting these pumps significantly reduces the concentration of antimicrobial required for treatment (Lomovskaya et al., 2001). Unfortunately, *P. aeruginosa* also has several other mechanisms of resistance, including decreased membrane permeability and enzymes that modify or degrade antimicrobials (Kon and Rai, 2016). Drug targets are also prone to mutate to avoid antimicrobial sensitivity. In addition, *P. aeruginosa* produces robust biofilms that form a physical barrier between the pathogen and antimicrobial therapies (Mah and O’Toole, 2001; Anderson and O’Toole, 2008). *P. aeruginosa* readily acquires genetically encoded resistance determinants from other pathogens, further increasing resistance. The development and approval of new antimicrobials (especially new classes of antimicrobials) is typically a slower process than the development of resistance by the pathogen which also contributes to the increasing demand to at least supplement drug development efforts with novel treatment avenues for *P. aeruginosa* infections.

Perhaps the most promising route toward this goal is to target the virulence factors instead. Often, compromising these factors does not directly impinge on bacterial viability, which is predicted to reduce the pressure to evolve resistance. Virulence determinants come in a staggering array of forms, including toxins that directly damage the host (e.g., type III secretion effectors, pyocyanin, hemolysins, etc.) (Saliba et al., 2002; Cezairliyan et al., 2013; Managò et al., 2015; Dortet et al., 2018), proteins or co-factors that provide nutritional support for the pathogen (e.g., siderophores, carbohydrate permeases, etc.) (Minandri et al., 2016), or promote colonization (e.g., adherens, quorum-sensing, and biofilm formation). Small-molecule inhibitors that target these systems mitigate bacterial pathogenicity, facilitating bacterial clearance by the host. Based on this idea, a number of antivirulence compounds have been proposed for *P. aeruginosa*, mostly targeting the pathogen’s quorum-sensing systems and biofilm-forming capacity (Vandeputte et al., 2011; Tan et al., 2013; Imperi et al., 2014; Kang and Kirienko, 2017; Paczkowski et al., 2017; van Tilburg Bernardes et al., 2017; D’Angelo et al., 2018).

Although acute virulence determinants have generally received less attention in recent studies, the siderophore pyoverdine makes an attractive therapeutic target for several reasons. First, pyoverdine is essential for *P. aeruginosa* pathogenesis in various mammalian and invertebrate host models (Meyer et al., 1996; Takase et al., 2000; Imperi et al., 2013; Kirienko et al., 2013; Lopez-Medina et al., 2015; Minandri et al., 2016). Pyoverdine’s exceptionally high affinity for ferric iron allows it to scavenge trace iron from the environment and also to abstract it from host iron-sequestering proteins such as transferrin and lactoferrin (Meyer et al., 1996; Xiao and Kisaalita, 1997). Second, pyoverdine can also hijack iron from other host sources, including mitochondria, which inflicts considerable damage on the host (Kirienko et al., 2013, 2015; Kang et al., 2018). Third, iron-bound pyoverdine (known as ferripyoverdine) functions as a signaling molecule that triggers the release of the alternate sigma factor PvdS from sequestration by the intermembrane FpvA/FpvR complex (Beare et al., 2003). Once released, PvdS promotes the expression of at least two secreted toxins (the translational inhibitor ToxA and the protease PrpL) and also its own biosynthetic machinery (Ochsner et al., 1996; Wilderman et al., 2001; Lamont et al., 2002). Fourth, the iron provided by pyoverdine is required for biofilm formation (Banin et al., 2005). Finally, and perhaps most importantly, fluoropyrimidines have been successfully used in several hosts to target pyoverdine, which has substantially reduced host mortality (Imperi et al., 2013; Costabile et al., 2016; Kirienko et al., 2016).

In this study, we describe the identification of a group of novel small molecules that compromise pyoverdine function without acting as conventional antimicrobials. We show that these compounds ameliorate pyoverdine-mediated damage, and that their effect is specifically due to their inhibition of pyoverdine function. These drugs rescue *Caenorhabditis elegans* at mid-micromolar concentrations and exhibit drug-like profiles. Promisingly, these compounds demonstrate synergy when combined with a conventional anti-Pseudomonal antibiotic. These compounds may serve as drug leads themselves or tool compounds for the identification of a future class of virulence inhibitors with clinical utility.

## Materials and Methods

### Bacterial and *C. elegans* Strains and Maintenance

The strains used in this study can be found in Table 1. *C. elegans* strain SS104 [*glp-4(bn2)*] was maintained on nematode growth medium (NGM) seeded with *Escherichia coli* strain OP50 at 15°C (Stiernagle, 2006). *P. fluorescens* WCS365 (Geels and Schippers, 1983) was a gift from Dr. Cara Haney, and was selected as a high pyoverdine producer. The *P. aeruginosa* PA14*pvdF* mutant has a Mariner transposon inserted into the *pvdF* locus, as verified by Sanger sequencing (Liberati et al., 2006). *Enterococcus faecalis* was a gift from Dr. Danielle Garsin.

**Table 1:** Strains used in this study.

Species	Strain	Reference
*C. elegans*	*glp-4(bn2)*	Beanan and Strome, 1992
*E. coli*	OP50	Stiernagle, 2006
*P. aeruginosa*	PA14	Rahme et al., 1995
*P. aeruginosa*	PA14Δ*pvdA*	Shanks et al., 2006; Kuchma et al., 2007
*P. aeruginosa*	PAO1	Holloway et al., 1994
*P. aeruginosa*	Boston 41501	ATCC 27853 (Medeiros et al., 1971)
*P. aeruginosa*	KM 306	ATCC 25010 (Yabuuchi and Ohyama, 1972)
*P. aeruginosa*	6092	ATCC 33360 (Liu et al., 1983)
*P. fluorescens*	WCS365	Geels and Schippers, 1983
*E. faecalis*	OG1RF	Garsin et al., 2001


### *Caenorhabditis elegans* – *P. aeruginosa* Assay

The Liquid Killing assay was performed essentially as described (Anderson et al., 2018). In brief, *glp-4(bn2ts)* worms were synchronized by bleaching gravid adults and hatching in the absence of food at 15°C. L1 larvae were dropped onto plates containing standard *E. coli* OP50, and then transferred to the non-permissive temperature to induce sterility. At the young adult stage, they were washed from plates, and sorted into 384-well plates containing *P. aeruginosa*. The bacteria were prepared by spreading a small amount of an overnight inoculum onto an SK media plate (Kirienko et al., 2014), and incubating the plate at 37°C for 1 day. Bacteria were then scraped from the plate and inoculated into LK media at a final OD_600_ of 0.03.

For the assessment of potential synergistic interaction between antimicrobials and anti-virulents, *C. elegans* were incubated with *P. aeruginosa* strain PA14 for 24 h, After that 200 μg/mL (final) of gentamicin with or without pyoverdine inhibitors (100 μM) was added. Host survival was examined 24 h later using Sytox Orange staining as per standard Liquid Killing protocol.

### High-Throughput Screening Hits

A high-content, high-throughput screen using the Liquid Killing assay was previously performed as described (Kirienko et al., 2016). Libraries with >1000 compounds screened are listed in Supplementary Table S1. Screening hits were purchased from varying companies through the chemical marketplace MolPort (Riga, Latvia). Small molecule purity (by HPLC) and identity (by mass spectrometry) was confirmed by vendors. When possible, compounds were sourced from more than one company to limit the possibility of misidentification. Analogous compounds were identified by searching chemical landscape using the Structure Search tools at MolPort.

### Production of Cell-Free, Pyoverdine-Rich Filtrate

To produce pyoverdine-rich filtrate, *P. aeruginosa* or *P. fluorescens* strains were grown in modified M9 medium [1% (w/v) Difco 5× M9 salts, 11.3 g/L casamino acids, 0.4% glucose, 1 mM CaCl2, 1 mM MgSO4] for 20–22 h at 28°C (*P. fluorescens*) or 37°C (*P. aeruginosa*) with agitation. Subsequently, bacteria were pelleted by centrifugation at 10,000 *g* for 30 min. Supernatants were sequentially filtered through 0.45, 0.20, and 0.20 μm filters to remove residual bacteria. The resultant cell-free, pyoverdine-rich material was referred to as filtrate throughout the manuscript. All steps were performed using plastic containers.

### Pyoverdine Measurements

A Cytation 5 multimode plate reader (BioTek Instruments, VT, United States) was used to assess quenching of pyoverdine by small molecules. To test pyoverdine fluorescence, *P. aeruginosa* strain PA14 was grown overnight in the presence of compounds, a single colony of *P. aeruginosa* strain PA14 was used to inoculate an overnight culture in LB, which was grown with agitation for 16 h at 37°C. The resulting culture was diluted 1000-fold and inoculated into modified M9 medium (see below) containing compound, DMSO, or FeCl_3_ at 100 μM. Cultures were incubated at 37°C with shaking for 24 h. The resulting cultures were centrifuged, and pyoverdine fluorescence in the media was determined spectrophotometrically with excitation 405 nm and emission 460 nm. Similarly, the ability of the compounds to quench pyoverdine fluorescence in bacteria-free pyoverdine-rich filtrate was assayed by adding equal amounts of bacteria-free, pyoverdine-rich filtrate and modified M9 medium containing 100 μM compound, DMSO, or FeCl_3_. Quenching was measured after a 5 min incubation at room temperature. All pyoverdine measurements were performed within the linear range of the Cytation 5, which was empirically determined to be between 1,000 and 20,000 arbitrary fluorescence units using serial dilutions of pyoverdine-rich cell-free filtrate.

### RNA Purification, NanoString, and qRT-PCR

For NanoString (NanoString Technologies, WA, United States) studies, 2,000 worms were exposed to 100 μM of LK11, LK31, LK31a, or ciclopirox olamine in S Basal media for 16 h in 6-well plates. Worms incubated in corresponding amount of DMSO for 16 h served as a normalization control. Afterward, worms were transferred into 15 mL conicals and washed twice with S Basal. Two biological replicates were tested for each condition. Gene expression was analyzed according to NanoString guidelines. For experiments involving quantification of expression of pyoverdine-dependent genes in the host, *glp-4(bn2)* worms were exposed to pyoverdine for 16 h in the presence or absence of small molecules at 50 μM. RNA collection and qRT-PCR were performed as previously described (Kirienko et al., 2008). Fold-changes were calculated using a ΔΔCt method. Primer sequences are available upon request.

For assessing the effect of LK31 and LK31a on the expression of pyoverdine-dependent genes in *P. aeruginosa*, experiments were performed as follows: after 14 h growth in 6 mL of SK media supplemented with either DMSO or 100 μM inhibitor, cells were collected via centrifugation. RNA was extracted and purified using TRIzol reagent (Invitrogen, CA, United States) according to the manufacturer’s protocols with minor adjustments. To ensure cell lysis, cells resuspended in TRIzol reagent were freeze-cracked and vortexed prior to phase separation. Purified RNA was treated with DNase I (Thermo Fisher Scientific, MA, United States) to eliminate genomic DNA contamination. Reverse transcription was performed using random decamers and Retroscript Kit (Thermo Fisher Scientific, MA, United States). qRT-PCR was conducted using SYBR green AzuraQuant Fast Green Fastmix (Azura, MA, United States) in a CFX-96 real-time thermocycler (Bio-Rad, CA, United States). Fold-changes were calculated using a ΔΔCt method. Primer sequences are available upon request.

### Compounds’ Effect Under Iron-Limiting Conditions

WT *P. aeruginosa* PA14 or the PA14Δ*pvdA* mutant were grown in 96-well plate in M9 media supplemented with casamino acids (BD Bacto, CA, United States) and with or without 6.25 μM ciclopirox olamine (CPX). PA14 CPX samples were supplemented with either DMSO or pyoverdine inhibitors LK11, LK31, and LK31a at 100 μM. The 96-well plate was incubated in Cytation 5 multimode plate reader (BioTek, VT, United States) at 37°C. OD_600_ readings were recorded every 30 min for 22.5 h. Three biological replicates were performed.

### MIC, CFU, and Microscopy Assays

To determine the minimum inhibitory concentration (MIC) of compounds for preventing bacterial growth, *P. aeruginosa* strain PA14 was grown in standard LB overnight and diluted 100,000-fold in SK media. Compounds were two-fold serially diluted and mixed with equal volumes of diluted bacteria in 96-well plates. Plates were incubated at 37°C for 24 h. Growth inhibition was visually scored on the basis of turbidity. Two wells were used per condition, and at least three biological replicates were performed. For quantification of colony-forming units (CFU), shaking cultures of *E. coli* strain OP50, *P. aeruginosa* strain PA14, or *E. faecalis* strain OG1RF were grown at 37°C. At appropriate time points, aliquots were taken from each culture, serially diluted five-fold in S Basal, and plated onto LB agar. Colonies were counted under a dissecting microscope.

For assessing the impact of LK12 treatment on *P. aeruginosa* strain PA14, a strain carrying a plasmid encoding GFP was inoculated at an initial concentration of OD600 = 0.0001 in the presence of either 250 μM NK12 or DMSO alone (1% v/v). All cultures included propidium iodide at a final concentration of 40 μg/mL. At 4 or 8 h after inoculation, bacteria were removed and imaged using a Zeiss M2 upright fluorescent microscope (Carl Zeiss, Germany). Experiments with *Escherichia coli* strain OP50 transformed with a GFP-encoding plasmid were performed in the same fashion.

## Results

### Selected Hits That Rescue *C. elegans* From *P. aeruginosa* Interfere With Pyoverdine Fluorescence

**FIGURE 1:**
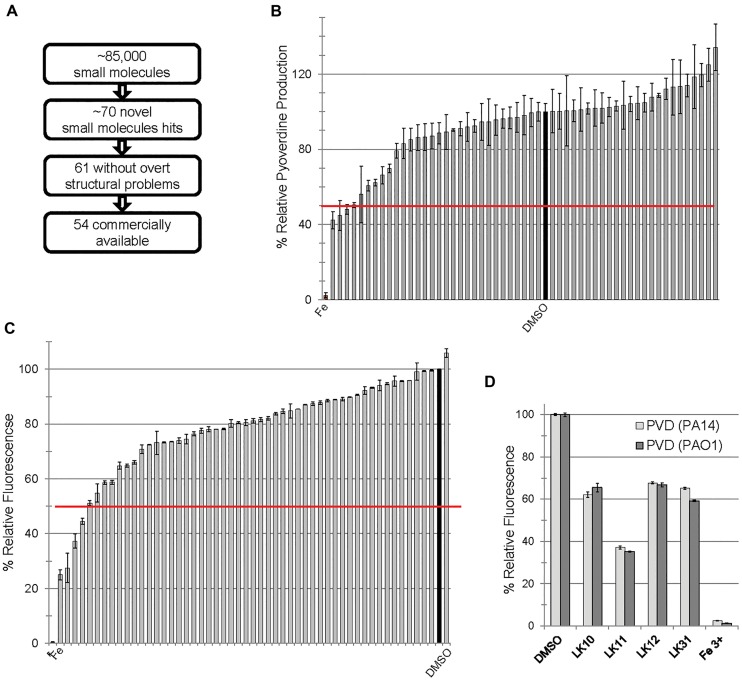
Small molecules interfere with pyoverdine function. **(A)** Flow chart of screening summary for novel small molecules that rescue *Caenorhabditis elegans* from *Pseudomonas aeruginosa*. **(B)** Effect of small molecule hits on fluorescence of pyoverdine when bacteria were cultured 24 h in the presence of the compounds (250 μM) or DMSO. **(C)** Fluorescence of pyoverdine in bacteria-free filtrate after a 5 min incubation with compound (250 μM). **(D)** Selected small molecules are capable of quenching pyoverdine fluorescence from pyoverdine-rich filtrates produced by *P. aeruginosa* strain PA14 or PAO1. At least three biological replicates were performed for **B–D**. The red lines in **B,C** correspond to 50% decrease in fluorescence.

We previously carried out a fragment-based, high-content, high-throughput phenotypic screen comprising over 86,000 wells (Kirienko et al., 2016). The goal was to identify compounds that increase *C. elegans* survival during exposure to *P. aeruginosa* strain PA14. This assay identified approximately 70 novel small molecules that passed initial and second-pass retesting that showed statistically significant rescue. Apparent pan-assay interfering compounds (Baell and Holloway, 2010; Baell and Walters, 2014) were removed, leaving 61 compounds, 54 of which were commercially available at the time of our initial retesting (Figure 1A). We have previously demonstrated that some of the hits acted by interfering with bacterial iron metabolism (Kirienko et al., 2013) or by inhibiting the production of pyoverdine (Kirienko et al., 2016). Since pyoverdine toxicity is the principal factor underlying host killing, we hypothesized that at least some of the remaining hits may act by blocking pyoverdine function.

In the past, we have observed a strong positive correlation between pyoverdine fluorescence and toxicity (Kirienko et al., 2013; Kang et al., 2018). On this basis, we predicted that we would be able to identify compounds that compromise pyoverdine toxicity by measuring fluorescence in the presence of the compound. We tested the ability of each of the 54 commercially available hits to reduce pyoverdine fluorescence in growing cultures of *P. aeruginosa* PA14 (Figure 1B). We also added each of the commercially available compounds to pyoverdine-enriched, cell-free filtrates to verify that a reduction in fluorescence would be observed there as well, to rule out the possibility that the compounds were merely preventing pyoverdine production or were non-specifically compromising bacterial growth. Using these assays we identified four hits (LK10, LK11, LK12, and LK31) that quenched more than half of pyoverdine fluorescence (Figure 1C). These compounds also dramatically reduced pyoverdine fluorescence in *P. aeruginosa* PAO1, another clinically derived strain (Figure 1D).

**FIGURE 2:**
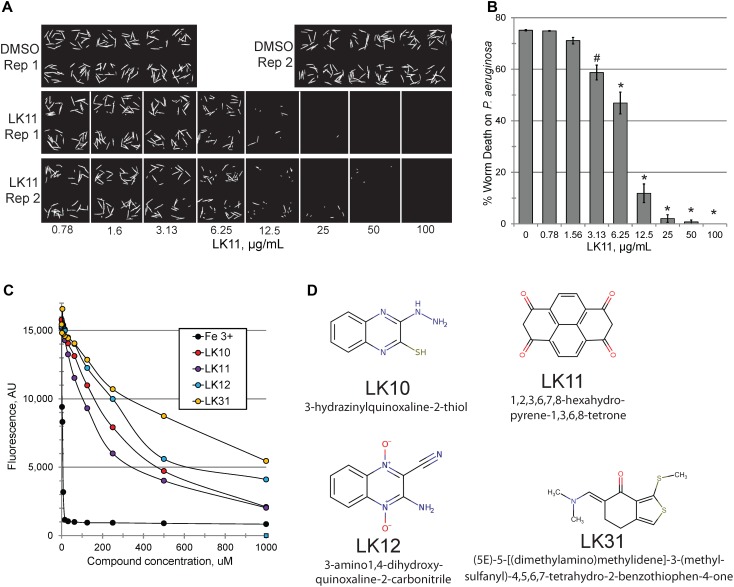
LK11 demonstrates dosage-dependent rescue. Representative images **(A)** and quantification **(B)** of *C. elegans* death after incubation with *P. aeruginosa* in the presence of varying concentrations of LK11. The cell-impermeable dye Sytox Orange was used to stain dead worms. Each well contains ∼18 worms. At least three biological replicates were performed. **(C)** Pyoverdine-rich filtrate was incubated with various concentrations of pyoverdine inhibitors or iron (III) and fluorescence was measured after 5 minutes. **(D)** The structures and IUPAC names of of LK10, LK11, LK12, and LK31. *p*-values were calculated using Student’s *t*-test, ^∗^*p* < 0.01, ^#^*p* < 0.05.

**FIGURE 3:**
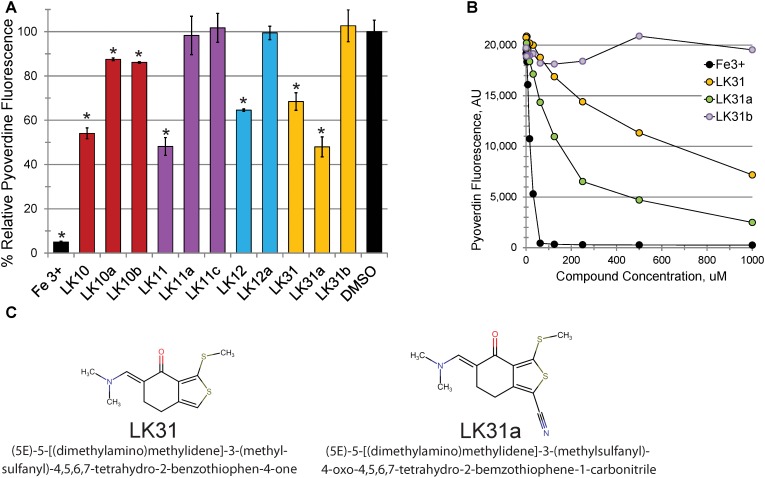
Selected hits show dosage-dependent pyoverdine fluorescence quenching. **(A,B)** Fluorescence of bacteria-free, pyoverdine-rich filtrate after 5 min incubation with the compounds indicated at either fixed (250 μM, **A**) or varying **(B)** concentrations. DMSO. **(C)** Structural comparison of a parent compound (LK31) and an analog with improved efficiency (LK31a). At least three biological replicates were performed for **A,B**. *p*-values were calculated using Student’s *t*-test, ^∗^*p* < 0.01.

Consistent with our predictions, the ability of the compounds to both rescue *C. elegans* (example shown in Figures 2A,B) and quench pyoverdine (Figure 2C) were dosage dependent. Interestingly, the four pyoverdine inhibitors represented diverse structural classes (Figure 2D). In an effort to better relate the structures and the activities of the compounds, we searched for commercially available analogs. We used a structure-based search tool to identify the closest commercially available compounds, and acquired up to three analogs for each (see Supplementary Figure S1 for structures). Each analog was solubilized in DMSO and tested for the ability to reduce pyoverdine fluorescence (Figures 3A,B). LK31a, which differs from its parent compound LK31 by the addition of a carbonitrile group (Figure 3C), showed significantly greater efficacy in this assay than its parent molecule. In contrast, LK31b had no discernible activity. None of the analogs selected for LK10, LK11, or LK12 exhibited anti-pyoverdine activity greater than their parent compounds.

**FIGURE 4:**
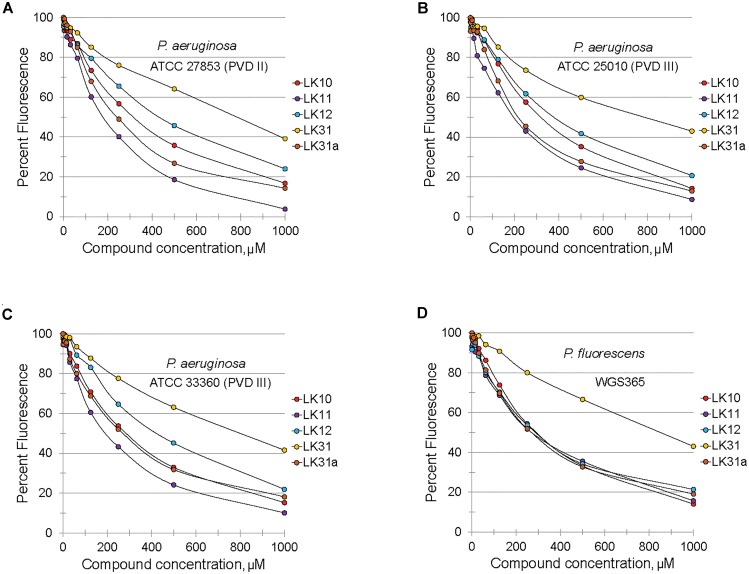
Selected hits inhibit fluorescence of structurally diverse pyoverdines. **(A–D)** Fluorescence of bacteria-free, pyoverdine-rich filtrate after 5 min incubation with compounds at varying concentrations or DMSO. Filtrates were produced from the indicated strains. At least two biological replicates were performed.

Structurally, pyoverdine is comprised of two disparate parts. The chromophore, which is shared by all fluorescent Pseudomonads, consists of a relatively compact, heavily modified dihydroxyquinoline moiety. Attached to this is an oligopeptide chain that is produced by non-ribosomal protein synthesis machinery and introduces a variety of non-standard and *D*-conformation amino acids into the chain. The genes involved in biosynthesis of the oligopeptide side chain exhibit greater sequence variation than most regions of the *P. aeruginosa* genome. As a consequence, Pseudomonads produce pyoverdines that are sufficiently variable for use as a tool for taxonomic differentiation, a process called siderotyping (Meyer et al., 1998; Fuchs et al., 2001; Meyer et al., 2002). Early in the molecular study of pyoverdine, when it became apparent that pyoverdines had heterogeneous amino acid chains, they were designated as type I, type II, and type III (Meyer et al., 1997; De Vos et al., 2001). Since clinically relevant strains of *P. aeruginosa* are known to produce each of these types of pyoverdine, we obtained relevant strains of *P. aeruginosa* from the ATCC. We also tested compound efficacy against pyoverdine produced by *P. fluorescens*, which is likely to be even more variant from the type I producing strains we already tested (PAO1 and PA14). In each case, the compounds quenched pyoverdine fluorescence (Figure 4).

### LK10 and LK12 Are Multifunctional Compounds

**FIGURE 5:**
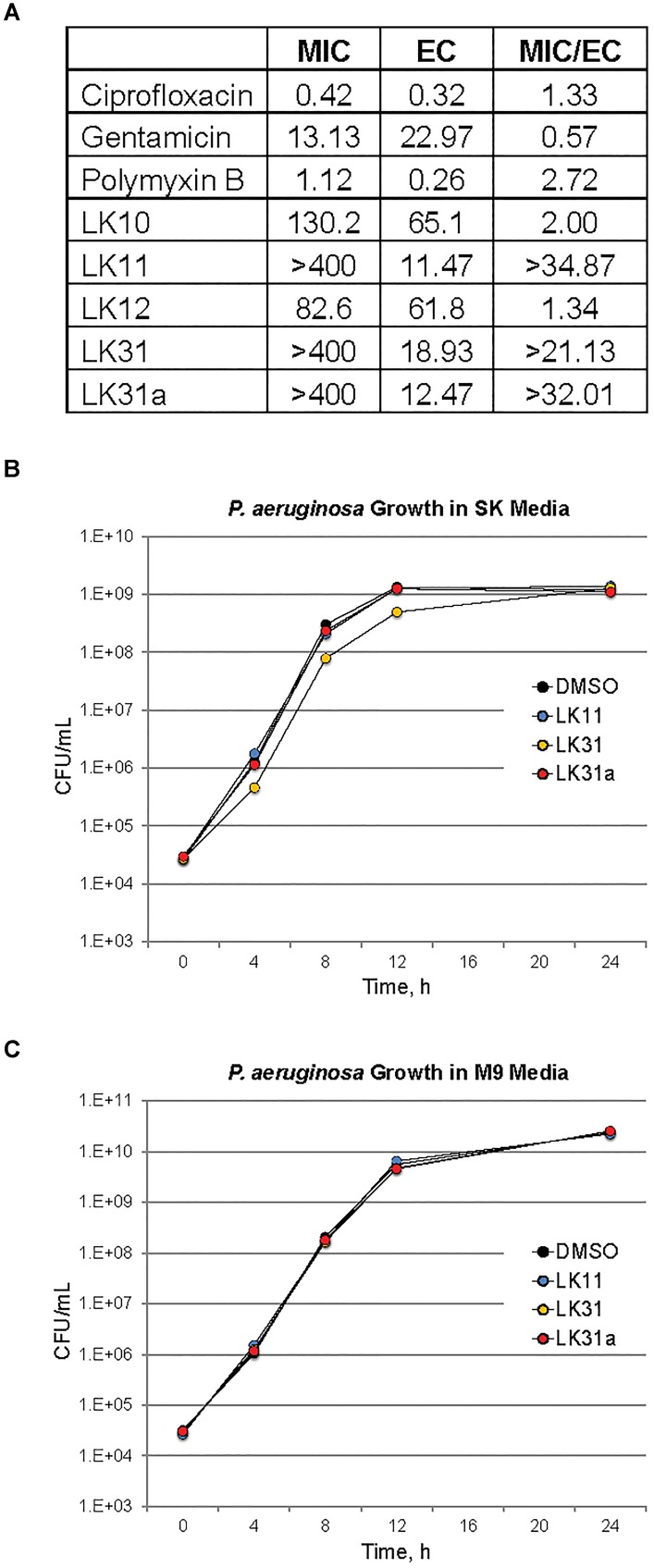
A subset of pyoverdine inhibitors behave as anti-virulents. **(A)** MICs, effective concentrations (EC, defined as the minimum concentration required for statistically significant amelioration of killing), and the ratios of MIC to EC are shown for a panel of classical antimicrobials, the antivirulents LK11, LK31, and LK31a, and the bifunctional compounds LK10 and LK12. **(B,C)** Colony-forming unit counts for *P. aeruginosa* grown in the presence of DMSO or 100 μM of LK11, LK31, and LK31a in nutrient-poor SK media **(B)** or more nutritious M9 media **(C)**. At least three biological replicates were performed for each experiment.

Although LK10, LK11, LK12, and LK31 reduce pyoverdine fluorescence, we tested whether the compounds also act as antimicrobials by examining their ability to prevent bacterial growth. Compounds were serially diluted in 96-well plates and *P. aeruginosa* was added and grown overnight. We also tested several standard antimicrobials with efficacy against *P. aeruginosa* as controls. Compounds with MIC values similar to their effective rescuing concentrations (defined as the concentration required for statistically significant rescue in Liquid Killing) were considered to have antimicrobial activity. For comparison, the known antimicrobials we tested showed MIC/EC ratios between 0.5 and 3 (Figure 5A). LK10 and LK12 also had MIC/EC ratios close to this range. On this basis, we have reclassified them as hybrid molecules, as they both inhibit *P. aeruginosa* growth and also quench pyoverdine fluorescence.

Since LK12 demonstrated the lowest MIC (suggesting the strongest antimicrobial effect), we further examined its antibacterial properties. We grew *P. aeruginosa* in the presence of varying concentrations of LK12 and observed a concentration-dependent bactericidal effect (Supplementary Figure S2A). Interestingly, this effect appeared transient, as bacterial growth quickly recovered and the titer of viable bacteria was high by 16 h. We tested whether the compound also affected *E. coli* or *E. faecalis*. We noted that, although the compound had a more pronounced effect on *E. coli*, it was still transient (Supplementary Figure S2B). We hypothesize that the difference in efficacy is related to the difference in efflux pump efficiency and expression between *P. aeruginosa* and *E. coli.* Compound treatment slowed *E. faecalis* growth, but there were no overt signs that it was bactericidal, even at twice the concentration used for the gram-negative bacteria. This may suggest that LK12 is more effective in blocking the growth and development of gram-negative bacteria.

To verify that the bacteria were dead, we also tested *P. aeruginosa* PA14 that constitutively express GFP. Bacteria were grown in the presence of the compound for 8 h, and then washed and stained with propidium iodide. Propidium iodide is a cell-impermeant dye, and will stain only dead bacterial cells (Supplementary Figures S2C,D). After 8 h, a large proportion of bacteria showed staining. Interestingly, we observed a very different phenomenon when we tried an analogous experiment using *E. coli* strain OP50. Instead of dividing properly, the morphology of *E. coli* cells changed to a longer, more filamentous state (Supplementary Figures S2E,F). This is consistent with a bacterial stress response to DNA damage (Suzuki et al., 1967; Walker and Pardee, 1968; Cushnie et al., 2016). Our findings are consistent with earlier reports about the capacity of LK12 to generate ROS and induce DNA damage (Ismail et al., 2010; Cheng et al., 2016).

### Anti-pyoverdines Have Drug-Like Properties

We used the ability of LK11, LK31, and LK31a to quench pyoverdine fluorescence to determine their affinity for pyoverdine (Supplementary Table S2). Although the affinity of the compounds is relatively low, in the hundred micromolar range, this is common for ‘very promising’ hits from fragment-based drug discovery methods (Murray and Rees, 2009). It is worth noting that no overt toxicity was seen when *C. elegans* was exposed to 128 μM LK11, LK31, or LK31a (Supplementary Figure S3). The other traits of the compounds generally conform to common drug design guidelines, such as Lipinski’s Rule of Five (Lipinski et al., 2001) or The Rule of Three (Congreve et al., 2003; Table 2). These ‘rules’ have been empirically derived from successful drugs with known oral bioavailability and typically discriminate toward smaller, less reactive molecules. Consistent with this, all three have relatively low molecular weight (approximately 150–250 Da, Table 2). While not essential, low initial mass for leads is useful; drug development frequently involves adding functional groups that increase both affinity and mass. Based on these characteristics, LK11 and LK31/LK31a may serve as promising compounds for optimization. One intriguing possibility is that LK11 and LK31/LK31a could be combined into a single molecule with an even higher affinity if their binding sites are different and proximal to one another (Doak et al., 2016).

**Table 2:** Chemical properties of pyoverdine inhibitors.

				H Bond	H Bond	Rotatable
Molecule	MW, Da	LogP	Polar area	donor	acceptor	bonds
LK11	264.2	2.1	68.3	0	4	0
LK31	253.4	2.9	73.8	0	4	2
LK31a	278.4	2.9	97.6	0	5	2
Goal	<500	-0.4…5.6	<1	<5	<10	<10


### Pyoverdine Inhibitors Behave as *Bona Fide* Anti-virulents

Two factors suggested that LK11 and LK31 were acting as antivirulents. First, we were unable to use the compounds alone to inhibit bacterial growth in standard MIC assays. Second, the MIC/EC ratios of LK11 and LK31 were at least an order of magnitude higher than the ratios for known antimicrobials. As pyoverdine is a large molecule requiring substantial cellular investment, disrupting or delaying bacterial growth significantly will delay pyoverdine biosynthesis. To rule out this potentially confounding issue, we carefully compared bacterial growth for cultures with various concentrations of LK11, LK31, LK31a, or DMSO alone. Rather than rely on spectroscopic measurements, which can be misleading, we serially diluted bacterial inocula and counted the number of viable bacterial colonies. Bacterial growth kinetics were unaffected by the presence of LK11, LK31, or LK31a in two different growth media (Figures 5B,C).

**FIGURE 6:**
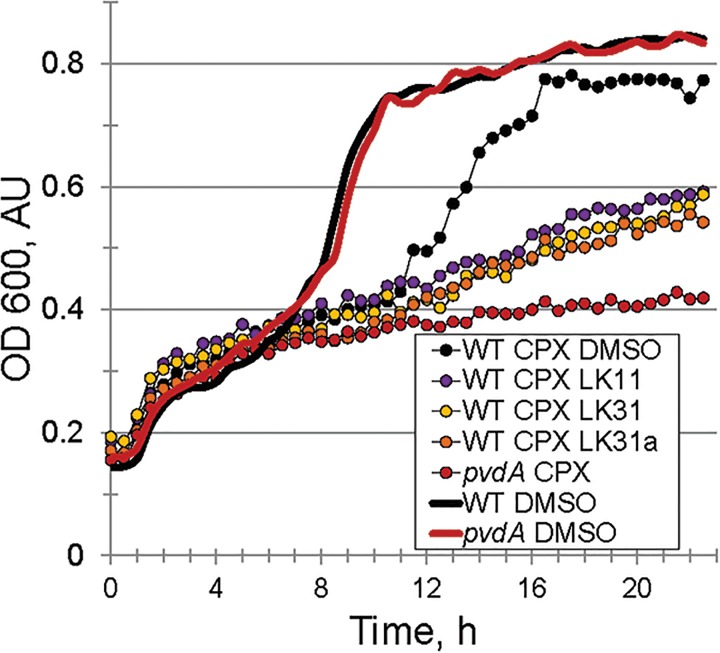
Pyoverdine inhibitors disrupt bacterial growth under iron-limiting condition. Growth curves of WT *P. aeruginosa* PA14 and PA14*pvdA*. Iron limitation was induced by the presence of 6.5 μM ciclopirox olamine (CPX), an iron (III) chelator. DMSO or compounds were added at 100 μM. Three biological replicates were performed.

Under iron-limiting conditions pyoverdine becomes essential for bacterial survival and growth. For this reason, we anticipated that the compounds may compromise bacterial growth under these conditions. First, we compared the growth of wild-type *P. aeruginosa* PA14 and Δ*pvdA* pyoverdine production mutant in M9 media supplemented with casamino acids. In the absence of an exogenous iron chelator, growth of the strains was indistinguishable (Figure 6). In contrast, the addition of 6.25 μM ciclopirox olamine (CPX) caused a small lag in wild-type development but compromised the growth of the Δ*pvdA* mutant, indicating that these conditions require pyoverdine for growth. Adding LK11, LK31, or LK31a to wild-type *P. aeruginosa* growing in the presence of CPX phenocopied the Δ*pvdA* defect, providing another piece of evidence to support that the compounds are compromising pyoverdine function.

Although these data substantially demonstrated that the pyoverdine inhibitors were compromising pyoverdine fluorescence and that they were not functioning by preventing bacterial growth under iron replete conditions, we also wanted to eliminate the possibility that their effect was due to the compounds exerting some uncharacterized effect on the host. For example, previous reports have shown that small molecules can trigger host defenses and extend *C. elegans* survival, even if they are toxic and will eventually kill the host as well (Pukkila-Worley et al., 2012). To test this, we used NanoString technology to assess the mRNA levels of 115 genes involved in *C. elegans* immune and stress response pathways. Gene expression was normalized to three housekeeping genes; (see Supplementary Table S3 for the list of genes and fold-changes). mRNA was purified from *C. elegans* treated with either LK11, LK31, LK31a, or DMSO (as a control) in the absence of *P. aeruginosa* or pyoverdine. LK11 had minimal effect on the host, with only 4 genes (3.5%) showing upregulation between 3- and 10-fold. LK31 and LK31a each upregulated less than 10% of the genes surveilled (and generally upregulated the same genes, which is consistent with their structural similarity) (Table 3). In contrast, ∼34% of the genes were upregulated in *C. elegans* exposed to the iron chelator ciclopirox olamine.

**Table 3:** Pyoverdine inhibitors do not trigger overt host defense response.

	LK11	LK31	LK31a	CPX
>3, <10	4	8	8	24
>10, <50	0	2	2	10
>50	0	0	0	5
Sum up	4	10	10	39
% Up	3.5	8.7	8.7	33.9


*CPX – ciclopirox olamine, iron chelator. The number shown represents the number of genes in each category. Sum up – total number of upregulated genes. % Up – percentage of upregulated genes (out of 115 total).*

The screening assay used automatic scoring, and host death was inferred on the basis of staining with the cell impermeant dye Sytox Orange. Therefore, we also ruled out the possibility that the compounds were somehow preventing the dye from staining dead worms (or that the anti-pyoverdine compounds were interfering with the fluorescence of the dye) by staining heat-killed worms in the presence of the compounds (Supplementary Figure S4). As expected, staining was unaffected.

### Anti-pyoverdine Compounds Specifically Minimize Pyoverdine Toxicity

**FIGURE 7:**
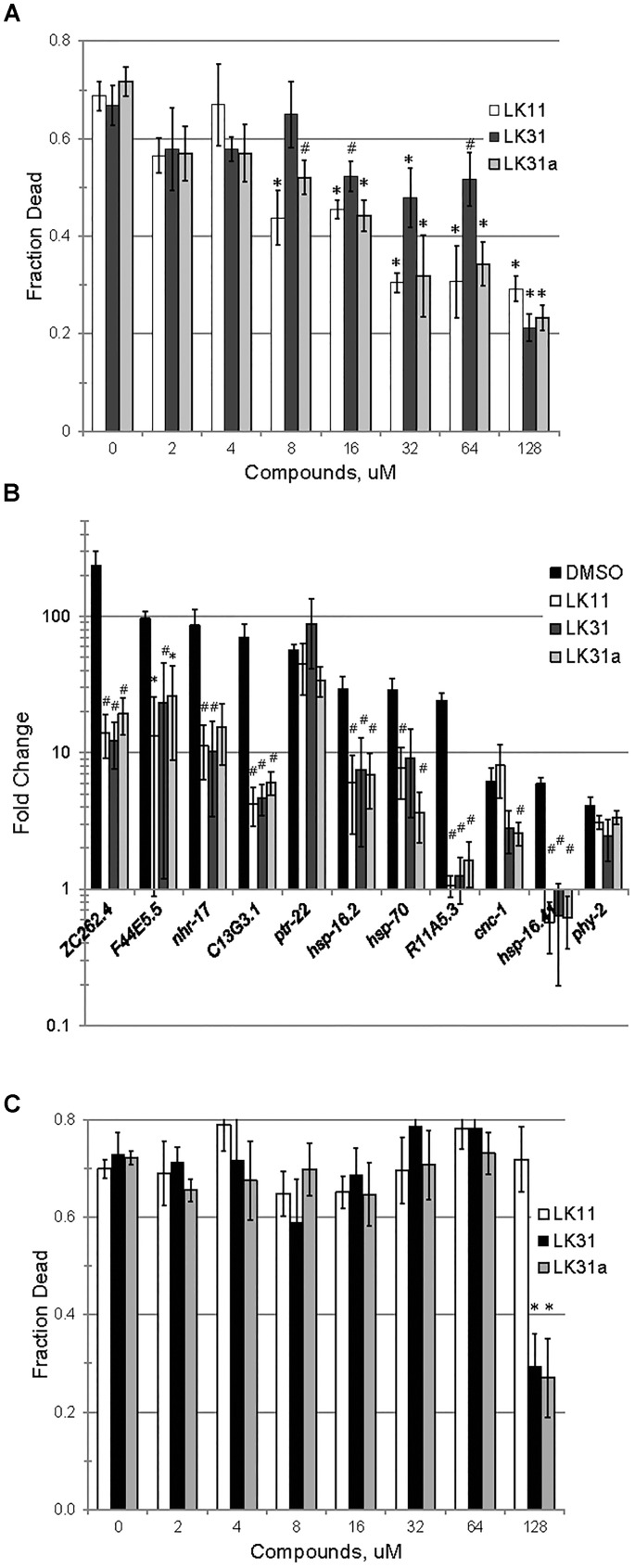
LK11, LK31, and LK31a inhibit pyoverdine-mediated pathology. **(A)** Liquid Killing of *C. elegans* was assayed in the presence of varying doses of LK11, LK31, and LK31a. **(B)** qRT-PCR showing expression of a panel of genes upregulated by pyoverdine exposure during Liquid Killing of *C. elegans* treated with LK11, LK31, or LK31a (100 μM). **(C)** Liquid Killing of *C. elegans* exposed to *P. aeruginosa* strain PA14*pvdF*, which is defective in pyoverdine biosynthesis, and treated with LK11, LK31, or LK31a at the concentrations indicated. At least three biological replicates were performed for **A–C**. *p*-values were calculated using Student’s *t*-test, ^∗^*p* < 0.01, ^#^*p* < 0.05.

We confirmed that LK11, LK31, and LK31a reduced bacterial virulence in *C. elegans*. As expected, all three compounds showed a strong, dose-dependent impact, limiting pathogenesis in the mid-micromolar range (Figure 7A). Based on our observations, these compounds are most likely functioning largely or entirely by limiting pyoverdine-mediated virulence. To further test this assertion, we measured the expression of pyoverdine-responsive genes (Kang et al., 2018) in *C. elegans* exposed to *P. aeruginosa* in the presence of LK11, LK31, LK31a, or DMSO. Upregulation of these genes was significantly diminished (Figure 7B) indicating that the toxicity of pyoverdine has been substantially blunted.

As noted above, iron-bound pyoverdine releases the alternative sigma factor PvdS from its sequestration at the plasma membrane, promoting the expression of several products, including itself, ToxA, and the proteast PrpL (Ochsner et al., 1996; Wilderman et al., 2001; Lamont et al., 2002). If the compounds in question are compromising pyoverdine function, we would expect transcription of these genes to be repressed in their presence. To test this, we assessed expression of PvdS-dependent genes (including *pvdS*, *pvdA*, *pvdE*, *pvdF*, *toxA*, and *prpL*) in *P. aeruginosa* PA14 grown in the presence of either DMSO, LK31, or LK31a, and observed that the compounds reduced their expression (Supplementary Figure S5). We also examined the expression of genes involved in pyochelin biosynthesis as a control. These genes were unaffected by treatment, as would be expected.

Although the anti-pyoverdine compounds LK11, LK31, and LK31a clearly quench pyoverdine fluorescence, reduce pyoverdine toxicity, and significantly decrease the pathogenesis of *P. aeruginosa*, it is formally possible that these phenomena are unrelated and that virulence attenuation is due to some other mechanism. The most straightforward way to test this is to assay whether the compounds mitigate *P. aeruginosa*-mediated virulence caused by a pyoverdine biosynthesis mutant. Pyoverdine mutants produce little to no pyoverdine and exhibit substantially reduced rates of killing *C. elegans* (Kirienko et al., 2013). We evaluated the ability of the *P. aeruginosa* PA14*pvdF* mutant strain to kill *C. elegans* when LK11, LK31, or LK31a are added. With the exception of the highest concentration tested (7- to 10-fold higher than the calculated EC), the compounds had little to no effect on the ability of the mutant strain to kill *C. elegans* (Figure 7C).

### Anti-pyoverdine Compounds Synergize With Antibacterial Agents

**FIGURE 8:**
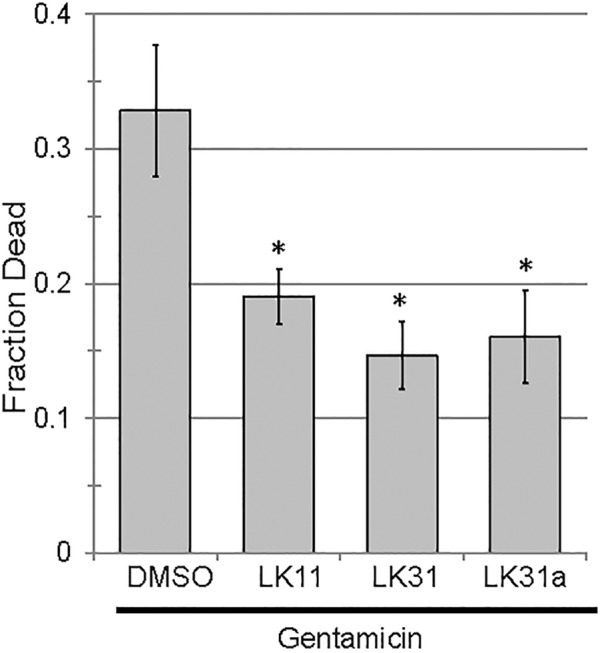
Pyoverdine inhibitors synergize with antimicrobial gentamicin. Liquid Killing of *C. elegans* exposed to *P. aeruginosa* for 24 h and subsequently treated with either gentamicin alone or gentamicin in combination with an antivirulent (100 μM). At least three biological replicates were performed. *p*-values were calculated using Student’s *t*-test, ^∗^*p* < 0.01.

In addition to serving as monotherapies, antivirulent drugs also have the potential to be included in therapeutic “cocktails” of multiple drugs that have synergistic activities. For example, the combination of pyoverdine inhibitors (to mitigate siderophore toxicity) and bacteriostatic or bactericidal drugs (to limit bacterial growth) is likely to be more effective than either drug alone. This is particularly due to pyoverdine’s ability to support other virulence factors, such as biofilms (reviewed in Kang and Kirienko, 2018). We predicted that the combination of an anti-pyoverdine like LK11, LK31, or LK31a with gentamicin would result in increased treatment efficiency in Liquid Killing when compared to antibiotic alone. To test this, we exposed worms to *P. aeruginosa* for 24 h and then added either antibiotic alone or antibiotic and an anti-pyoverdine compound. All three hit compounds showed a synergistic effect compared to antibiotic alone (Figure 8).

## Discussion

### Possible Mechanisms for Anti-pyoverdine Compounds

We observed that anti-pyoverdine compounds can quench the fluorescence of pyoverdine in cell-free filtrates, limit the expression of pyoverdine-dependent genes, and improve *C. elegans* survival after exposure to *P. aeruginosa* strain PA14. It is worth noting that, although they are effective at conferring resistance to pyoverdine, they do not completely abolish its activity, and are unlikely to compromise pyoverdine biosynthesis. This distinction is important for two reasons. First, non-vaccine treatments for bacterial pathogens need to be effective when used after infection. Second, *P. aeruginosa* recycles and reuses pyoverdine (Greenwald et al., 2007; Imperi et al., 2009). After import, oxidoreductases act upon ferripyoverdine to reduce iron (III) to iron (II), lowering the siderophore’s affinity for the metal. This facilitates the non-destructive removal of iron. Afterward, iron-free pyoverdine is exported to continue its activities, including inflicting additional damage and promoting toxin expression. For these reasons, preventing the biosynthesis of pyoverdine is less desirable than compromising its activity.

It remains an open question how LK11, LK31, and LK31a interact with pyoverdine. The simplest explanation for our observations is that these compounds bind to the conserved dihydroxyquinoline chromophore. This would explain the ability of the molecules to quench pyoverdine fluorescence, which is dependent upon this region of the molecule. It would also explain how the compounds can effectively quench the fluorescence of such disparate pyoverdines. Unfortunately, it is difficult to rule out the possibility that the compounds are binding the peptide sidechains. The short length of these chains (generally between 8 and 15 amino acids) may limit the biophysical interactions that generally drive protein folding. As such, these peptide sequences could exhibit conformational similarities despite differences in primary sequence.

These compounds also restrict the production of pyoverdine-dependent virulence factors, including exotoxin A, the protease PrpL, and pyoverdine itself. Although we have not formally demonstrated that the pyoverdine-compound complex is incapable of binding to the FpvA receptor or that it prevents the release of PvdS from subcellular sequestration, the qRT-PCR data indicate that PvdS-mediated transcription is significantly reduced after compound treatment (Supplementary Figure S5). The most parsimonious explanation for this is that the compounds have prevented one or more of these feed-forward steps. This demonstrates the power of anti-virulents and validates pyoverdine as a target, since even these non-optimized hits show considerable potential to alleviate virulence in this model. The next step will be to test their ability to limit pathogenesis in a more complex host.

The other matter that remains to be resolved is whether the compounds preclude pyoverdine from binding iron. As demonstrated with ciclopirox olamine, these compounds are likely to transition from anti-virulents to antimicrobials under strict iron limitation, which is likely to be the case in most hosts, including humans. Anti-virulents are very desirable because they are anticipated to reduce the evolutionary pressure to acquire resistance. As such, it is undesirable for the pathogen to transition to a state where they act as antimicrobials. Ultimately, however, this difference may be somewhat academic. Virulence is a complex trait, associated with the expression of a wide variety of genes and the establishment of a specific homeostatic state in the pathogen. Using small molecules to meddle with this state could easily disrupt the pathogen’s ability to survive or reproduce, with unpredictable consequences that are likely to depend upon the nature of the resistance that arises (Allen et al., 2014; Rezzoagli et al., 2018).

One possible outcome is that disrupting pyoverdine would stimulate increased production of alternative iron acquisition systems, like pyochelin. It is difficult to predict what effect this would have on virulence; typically, pyochelin is generally reported to be dispensable for virulence in mammalian systems, especially when pyoverdine biosynthesis is intact (Takase et al., 2000; Minandri et al., 2016).

A less likely, but not disproven, possibility is that the compounds are preventing pyoverdine from carrying out its other activities without compromising its ability to bind iron. For example, previous reports have shown that pyoverdine inflicts considerable damage on mitochondria and activates mitochondrial surveillance pathways (Kirienko et al., 2015; Tjahjono and Kirienko, 2017; Kang et al., 2018). This almost certainly requires that the siderophore traverses the plasma membrane, both to directly acquire the iron that it removes as well as to bring it back to the pathogen. This is consistent with prior data from our lab that showed the pyoverdine removed iron from the host (Kang et al., 2018). This directly contrasts with another iron chelator, phenanthroline, which caused similar damage by chelating iron, but did not leave *C. elegans*. If the compounds are interfering with the ability of the siderophore to enter or exit host cells, virulence would be compromised in Liquid Killing, but iron acquisition in normal media would probably be unaffected.

### Other Methods to Target Pyoverdine

The contributions of pyoverdine cytotoxicity to mammalian pathology remain important and unresolved, but pyoverdine clearly represents a valuable therapeutic target. To date, several approaches have been taken to target the pyoverdine siderophore system. Based on work by the Quax Lab (Nadal Jimenez et al., 2010), several groups have targeted PvdQ, which is required for pyoverdine maturation. The Gulick Lab carried out a high-throughput screen to identify small molecule inhibitors, and found a biaryl nitrile inhibitor of PvdQ (Drake and Gulick, 2011; Wurst et al., 2014). Other hits were identified by the Schreiber labs (Theriault et al., 2010) and a boronated alkyl chain by the Fast lab (Clevenger et al., 2013).

Interestingly, the biaryl nitrile inhibitor appears to synergize with a second class of pyoverdine inhibitors, fluoropyrimidines (Wurst et al., 2014). These compounds, including 5-fluorocytosine (5-FC) and 5-fluorouridine (5-FU) have been shown to compromise pyoverdine biosynthesis (Imperi et al., 2013; Costabile et al., 2016; Kirienko et al., 2016). Normally used as an antifungal, 5-FC has proven to be an effective method to inhibit *pvdS* expression (Imperi et al., 2013). This sigma factor is necessary for expression of the biosynthetic machinery that produces pyoverdine (Lamont et al., 2002). Suppression of *pvdS* in this fashion is sufficient to mediate rescue in *C. elegans* and in mice (Imperi et al., 2013; Kirienko et al., 2016). Interestingly, the mechanism of action remains unknown, but appears to require conversion of 5-FC to the well-known chemotherapeutic 5-fluorouracil (Imperi et al., 2013). From there, the drug is further metabolized to 5-fluorouridine (Kirienko et al., 2016). It remains unclear how this latter disrupts pyoverdine biosynthesis. Nevertheless, while fluoropyrimidines may serve as drugs of last resort for patients infected with pandrug-resistant *P. aeruginosa*, their profound cytotoxicity would appear to limit the potential of these therapies. Another widely explored approach is to directly target pyoverdine by adding gallium (III) to the system to compete with Fe^3+^. Although the ionic radii of Ga^3+^ and Fe^3+^ are nearly identical, and most proteins that use Fe^3+^ as a cofactor can incorporate Ga^3+^, gallium is redox inactive under biological conditions. Since the removal of Fe^3+^ from pyoverdine requires its reduction to Fe^2+^, Ga^3+^ becomes irreversibly bound to pyoverdine. Tests in animal models show that gallium can disrupt *P. aeruginosa* growth and inhibit biofilm formation (Kaneko et al., 2007; Banin et al., 2008; DeLeon et al., 2009). Unfortunately, Ga^3+^ has proven less effective in human serum, as elegant work by Paolo Visca’s group demonstrated that the combination of *P. aeruginosa* proteases and pyoverdine’s capacity for removing iron from host proteins facilitated pathogen growth, even in the presence of maximally achievable concentrations of Ga^3+^ (Bonchi et al., 2015). Although it remains an open question whether gallium will be more effective in human pulmonary environments, the promise of the early results is tempered by these findings. Some available evidence also suggests that gallium-bound pyoverdine still triggers the production of most pyoverdine-dependent virulence factors (Garcia-Contreras et al., 2014), although others have not seen this effect (Rezzoagli et al., 2018). Several other concerns, such as the immunosuppressive effect of Ga^3+^ and the difficulty of effectively using gallium as a treatment after the establishment of an infection (as opposed to pre-treatment or simultaneous treatment, as was used in most model assays) have also been expressed (Minandri et al., 2014).

Although none of the antivirulents described for *P. aeruginosa* are ready for clinical use, it is quite clear that new treatments, especially those that are not conventional antimicrobials, are urgently needed. Pyoverdine represents an invaluable target, due to its direct toxicity, position at the crux of nutrient acquisition, and regulation of virulence factors. Accordingly, it is likely that it will be an important target for chemical intervention. The antivirulents in this paper demonstrate proof of principle for targeting pyoverdine’s function, rather than its synthesis. They also represent promising fragments for lead generation and will serve as useful tool compounds for new *P. aeruginosa* discovery.

## Author Contributions

All authors designed and performed the experiments, analyzed the results, edited the manuscript, and were aware of this submission. NK supervised the project. DKi wrote the first draft. NK acquired funding.

## Conflict of Interest Statement

The authors declare that the research was conducted in the absence of any commercial or financial relationships that could be construed as a potential conflict of interest.
