# Supplementary material for: Novel Pyoverdine Inhibitors Mitigate Pseudomonas aeruginosa Pathogenesis
Source: Front Microbiol. 2019 Jan 9;9:3317. doi: 10.3389/fmicb.2018.03317 (PMC6333909; doi:10.3389/fmicb.2018.03317)
Supplement: Supplementary file 2 [file Data_Sheet_1.pdf]

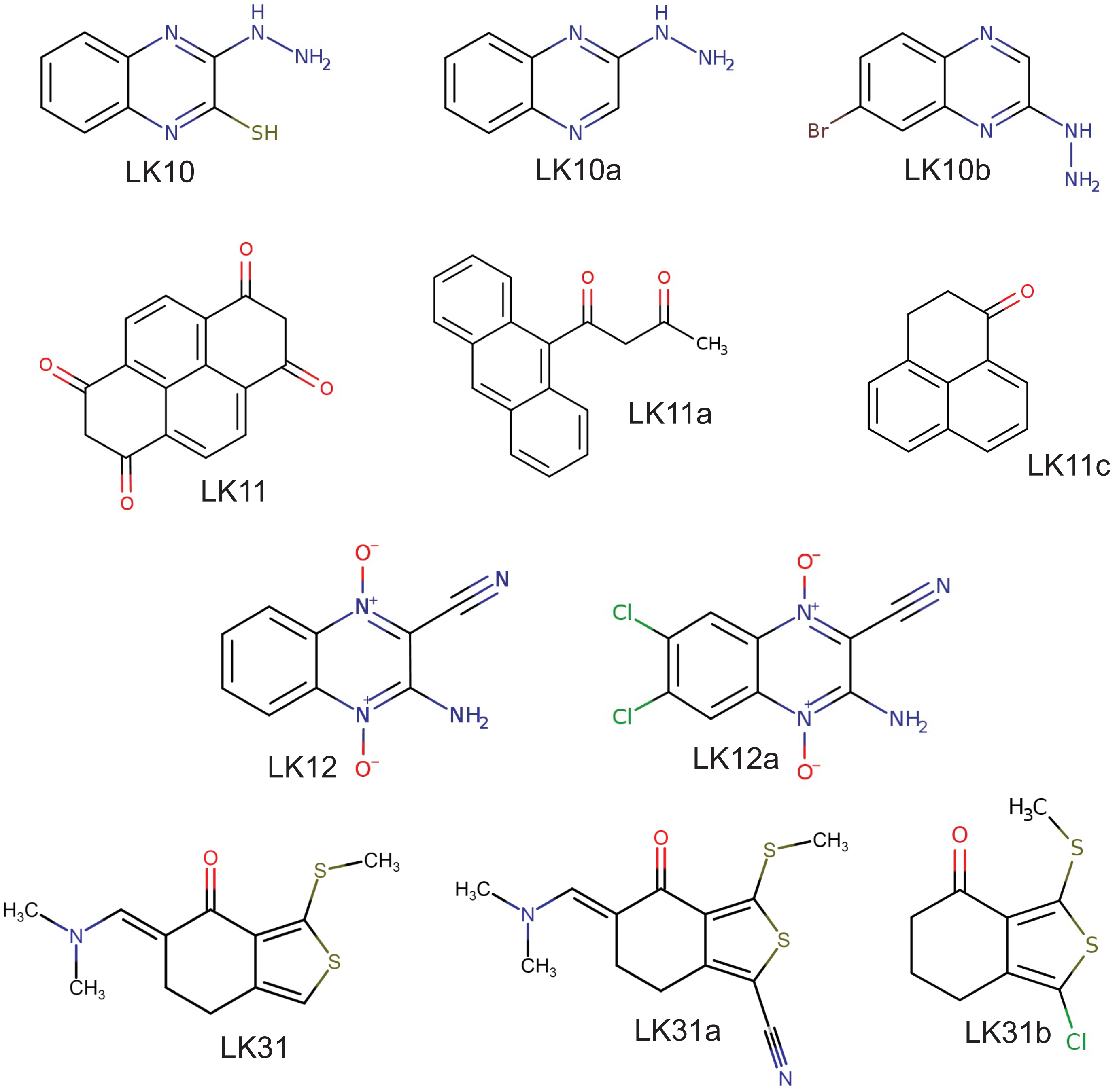

Figure S1. Structures of pyoverdine inhibitors and their analogs.

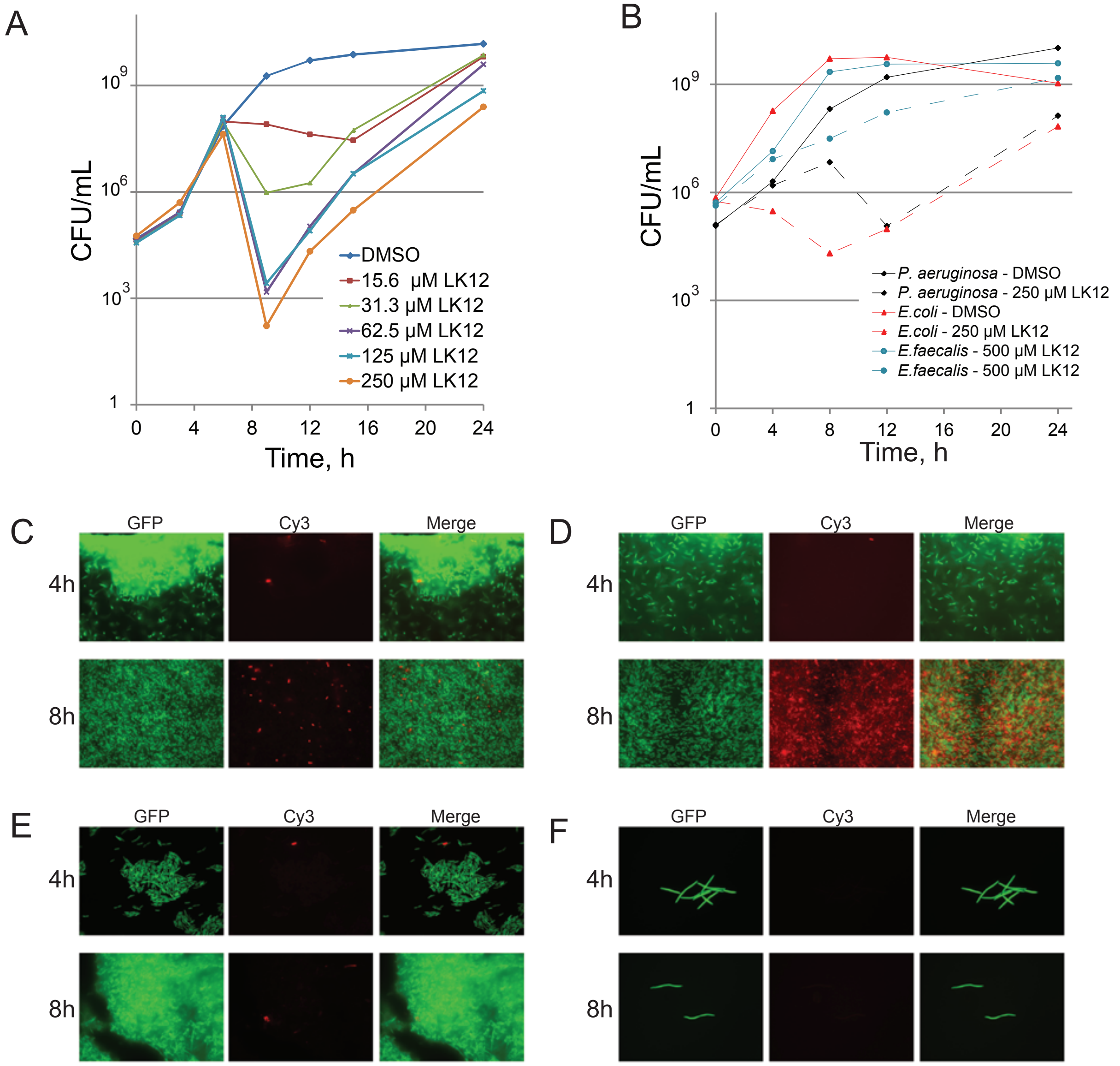

Figure S2. LK12 possesses antibacterial properties. (**A**) Growth curves of *P. aeruginosa* strain PA14 inoculated in LB in the presence of varying concentrations of LK12. (**B**) Effect of LK12 on growth of the gram negative bacteria *P. aeruginosa* and *Escherichia coli* or the gram positive pathogen *Enterococcus faecalis*. (**C-F**) Fluorescence microscopy of *P. aeruginosa* strain PA14-GFP (**C, D**) or *E. coli* (**E, F**) in the presence of DMSO (**C, E**) or 250 μM LK12 (**D, F**). In **C-F** dead cells were stained using the cell-impermeant dye propidium iodide. Leftmost panels in **C-F** illustrate bacterial GFP fluorescence, middle images are the Cy3 channel showing propidium iodide staining, and rightmost panels show merged fluorescence. At least three biological replicates were performed for each experiment.

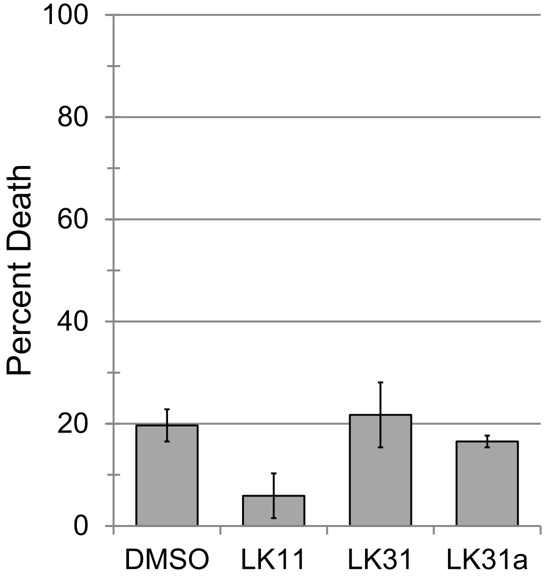

Figure S3. LK11, LK31, and LK31a do not cause overt host toxicity. *C. elegans* incubated for 7 days with DMSO or with 128 μM of LK11, LK31, or LK31a, and then stained with Sytox Orange, as per usual for Liquid Killing.

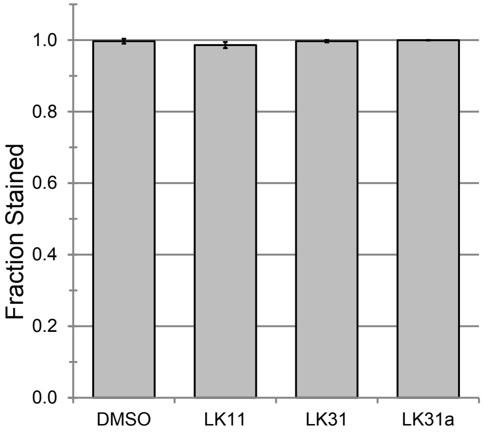

Figure S4. LK11, LK31, and LK31a do not interfere with Sytox Orange staining. Fluorescence of heat-killed *C. elegans*, incubated with 250 μM LK11, LK31, or LK31a for 8h prior to staining with Sytox Orange. 1 on Y axis corresponds to 100% of *C. elegans* staining, as expected for dead worms.

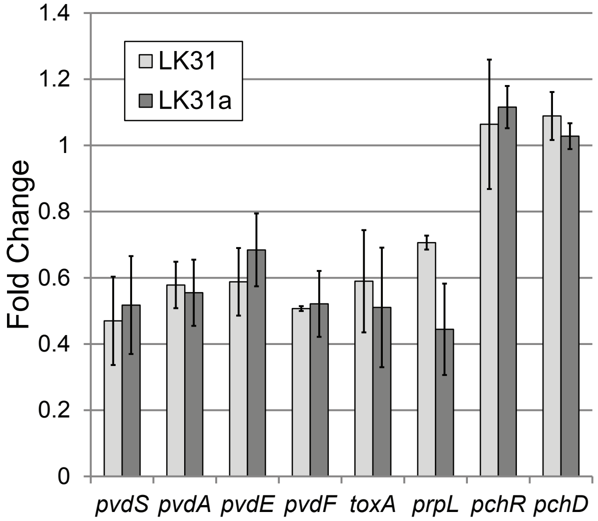

Figure S5. Expression of pyoverdine-dependent genes in the presence of LK31 and LK31a. qRT-PCR showing expression of a panel of known pyoverdine target genes and *pchD* and *pchR* in *P. aeruginosa* grown in the presence of DMSO or LK31, or LK31a (100 µM). Three biological replicates were performed.
