# Supplementary material for: Novel Pyoverdine Inhibitors Mitigate Pseudomonas aeruginosa Pathogenesis
Source: Front Microbiol. 2019 Jan 9;9:3317. doi: 10.3389/fmicb.2018.03317 (PMC6333909; doi:10.3389/fmicb.2018.03317)
Supplement: Supplementary file 3 [file Data_Sheet_2.pdf]

Table S1. Table of major small molecule libraries screened for the ability to alleviate *C. elegans* killing by *P. aeruginosa*.

Library Name	Cpds Screened
Prestwick 1 Collection	1020
Microsource - US Drug Collection	1020
LOPAC	1360
NINDS Custom Collection	1360
Life Chemicals	3872
Maybridge	6516
ChemBridge	10208
Asinex	12320
ChemDiv	14080
Enamine	24640

Table S2. Dissociation constants for LK11, LK31, LK31a and pyoverdine.

	Kd, μM
LK11	47
LK31	220
LK31a	104
